# Efficacy and safety of vedolizumab in elderly patients with inflammatory bowel disease: a matched case–control study

**DOI:** 10.1093/gastro/goz041

**Published:** 2019-09-17

**Authors:** Preeti Shashi, Dharmesh Gopalakrishnan, Malav P Parikh, Bo Shen, Gursimran Kochhar

**Affiliations:** 1 Center for Inflammatory Bowel Disease, Digestive Diseases and Surgery Institute, The Cleveland Clinic Foundation, Cleveland, OH, USA; 2 Department of Internal Medicine, The Cleveland Clinic Foundation, Cleveland, OH, USA; 3 Department of Gastroenterology, Allegheny General Hospital, Pittsburgh, PA, USA

**Keywords:** inflammatory bowel disease, vedolizumab, elderly patients, mucosal healing, safety

## Abstract

**Background:**

Vedolizumab was demonstrated to be safe and effective in adults with moderately to severely active inflammatory bowel disease (IBD) in clinical trials. However, there are limited data regarding its efficacy and safety in elderly patients.

**Methods:**

This was a case–control study comparing the efficacy (measured by rates of mucosal healing and need for IBD surgery) and safety of vedolizumab in IBD among patients ≥65 years of age (the elderly group) vs those <65 years (the control group). The two groups were matched individually on a 1:4 ratio based on gender and type of IBD. Conditional logistic regression was used for stratified analysis to calculate odds ratios and confidence intervals.

**Results:**

We included 25 IBD patients in the elderly group and 100 matched patients in the comparison group. Eighty patients had Crohn’s disease and 45 had ulcerative colitis. At baseline, the groups were comparable with regard to duration of IBD, prior anti-TNF therapy, and prior IBD surgery. The rate of mucosal healing on follow-up endoscopy was comparable between the elderly and control groups (50% vs 53%, *P *=* *0.507). Although more patients in the elderly group required IBD-related surgery while on vedolizumab, the difference did not reach statistical significance (40% vs 19%, *P *=* *0.282). Rates of vedolizumab-related adverse effects—rash, arthralgia, infections, infusion reactions, and dyspnea—were comparable between the two groups (all *P *>* *0.05).

**Conclusions:**

In a real-world setting, vedolizumab was demonstrated to have an efficacy and safety profile among elderly IBD patients that were comparable to younger controls.

## Introduction

Inflammatory bowel disease (IBD) is becoming more prevalent in the elderly due to the growing number of patients with long-standing or late-onset disease. It is estimated that 10%–15% of patients with IBD are diagnosed after the age of 60 years [1–5]. The management of IBD in elderly is particularly challenging due to medical co-morbidities, polypharmacy, physiological changes affecting drug delivery and metabolism, and higher risk of infections and malignancies with prolonged immunosuppressive therapy [6–9]. Moreover, elderly patients are hugely underrepresented in most clinical trials for biological therapies in IBD, limiting our understanding of their safety and efficacy in this population. Integrin receptor antagonists, by causing less systemic immunosuppression, may offer an attractive treatment option in these patients compared to TNFα antagonists or other immunosuppressive agents, though head-to-head studies are lacking.

Vedolizumab is a gut-selective humanized monoclonal IgG1 antibody that binds to α_4_β_7_ integrin, thereby modulating the trafficking of lymphocytes to the inflamed gut without interfering with their trafficking to other organs, including the brain [10–12]. It was demonstrated in phase III randomized placebo-controlled trials, GEMINI 1 and GEMINI 2, to be effective in inducing and maintaining clinical remission in patients with moderately to severely active ulcerative colitis (UC) and Crohn’s disease (CD), respectively, along with a favorable safety profile [13, 14]. However, these and subsequent vedolizumab trials enrolled few patients over the age of 65 years, and the available evidence regarding its safety and efficacy in the elderly is limited to post-hoc analyses. Moreover, studies that assessed the rates of mucosal healing in IBD with vedolizumab did not include patients older than 65 years. Our study aimed to directly compare the rates of mucosal healing and toxicities from vedolizumab among older (≥65 years) vs younger (<65 years) patients in a real-world setting.

## Methods

We conducted a retrospective electronic medical-records-based study with an individually matched case–control design. The therapeutic-efficacy and adverse-effect profiles of vedolizumab were compared between IBD patients 65 years or older (the elderly group) and those younger than 65 years (the control group).

### Inclusion and exclusion criteria

Twenty-five consecutive IBD patients ≥65 years of age at the time of first vedolizumab dose were included in the elderly group and 100 individually matched IBD patients, also on vedolizumab but <65 years of age at the time of first dose, were included in the control group. Consecutive elderly patients seen in our clinic were chosen irrespective of the type of IBD or previous therapies. Patients <18 years old were excluded from the study.

### Study design and baseline characteristics

Patients in the elderly group were matched individually on a 1:4 ratio, based on gender and type of IBD (CD or UC), to those in the control group. Information regarding baseline characteristics including age at IBD diagnosis and first vedolizumab dose, UC or CD phenotypes, previous IBD-related therapies, body mass index, smoking history, and baseline C-reactive protein were collected from electronic medical records.

### Outcomes

The primary efficacy outcome assessed was the rate of mucosal healing, as assessed by follow-up endoscopy. Mucosal healing on endoscopy was defined as the resolution of previously seen ulceration in CD or Mayo Clinic Endoscopic Subscore ≤1 in UC [15]. The resolution of previously observed bowel inflammation on follow-up CT or MR enterography was also assessed as a surrogate marker of efficacy. The need for IBD-related surgery while on vedolizumab for the treatment of medically refractory disease or complications was assessed as a secondary efficacy outcome. The incidence of the most frequent vedolizumab-related adverse events, namely arthralgia, rash, dyspnea, infections, and infusions reactions, were also compared between the two groups. Factors predicting the need for IBD surgery among the elderly were explored. Data regarding these outcomes were obtained retrospectively from electronic medical records.

### Ethical considerations

Approval for this research project was obtained from the Cleveland Clinic institutional review board. Requirement for informed consent was waived, since individual patient data were not published and were de-identified before analyses.

### Statistical analysis

Routine descriptive statistics including measures of central tendency were used. The SPSS software package (version 22.0) was used for statistical analyses. Conditional logistic regression for stratified analysis to calculate the odds ratio with 95% confidence intervals was used for the comparison of efficacy and toxicity outcomes between the two groups. The Fisher’s exact test was used to compare categorical variables while independent samples *t*-test was used to compare continuous variables. Kaplan–Meier time-to-event analysis was employed to compare time to surgery between the two groups, using the log-rank test. Logistic-regression models were used to assess for factors predicting the need for IBD-related surgery among elderly patients on vedolizumab. *P *<* *0.05 was considered to be significant.

## Results

### Baseline characteristics

We identified 25 patients in the elderly group and 100 matched patients in the control group. Sixty-four percent of patients in either group had CD and 52% were females. The mean age at initiation of vedolizumab was 69.5 years in the elderly group and 40.0 years in the comparison group (*P *<* *0.001). The groups were comparable at baseline with regard to IBD phenotypes, duration of disease, body mass index, smoking status, prior anti-TNFα therapy, and prior IBD surgery (Table 1).

**Table 1.** Comparison of demographic and other baseline characteristics

Characteristic	Age ≥65 years	Age <65 years	*P*-value
	(*n* = 25)	(*n* = 100)	
Age at IBD diagnosis, years	45.1 ± 7.2	24.8 ± 2.2	<0.001
Female gender	13 (52%)	52 (52%)	>0.999
Body mass index, kg/m²	25.6 ± 2.1	26.9 ± 1.4	0.289
Baseline CRP, mg/dL	0.95 (0.25–2.45)	0.75 (0.20–2.20)	0.808
Type of IBD			>0.999
CD	16 (64%)	64 (64%)	
UC	9 (36%)	36 (36%)	
UC-extensive disease	4/9 (44%)	22/29 (76%)	0.174
CD-ileocolonic disease	10/16 (62%)	51/64 (80%)	0.282
CD-stricturing	12/15 (80%)	48/62 (77%)	0.829
CD-penetrating	11/15 (73%)	39/62 (63%)	0.555
CD-perianal disease	6/15 (40%)	31/62 (50%)	0.572
Age at 1st Vedo dose, years	69.5 ± 8.4	40.0 ± 24.4	<0.001
Disease duration at 1st Vedo dose, years	23.7 (6.5–41.0)	16.1 (6.0–23.0)	0.893
Smoking (current or former)	15/25 (60%)	43/100 (43%)	0.178
Prior IBD surgery	15/25 (60%)	63/100 (63%)	0.820
Prior anti-TNFα	18/23 (78%)	89/98 (91%)	0.139

Data presented as mean ± standard deviation, median (interquartile range), or *n* (%).

IBD, inflammatory bowel disease; CRP, C-reactive protein; CD, Crohn’s disease; UC, ulcerative colitis; Vedo, vedolizumab.

### Efficacy outcomes

The median duration of follow-up after the first dose of vedolizumab was 7.4 months (interquartile range [IQR], 4.3–14.2 months) in the elderly group and 10.8 months (IQR, 5.3–15.0 months) in the control group. The rate of mucosal healing on follow-up endoscopy was comparable (50% in the elderly group vs 53% in the control group; *P *=* *0.507). Among patients who had evidence of bowel inflammation on baseline CT/MR enterography, the rates of resolution of these changes post vedolizumab were similar in the elderly and control groups (10% vs 15%; *P *=* *0.551) (Table 2). Though more patients in the elderly group required IBD-related surgery while on vedolizumab, the difference did not reach statistical significance (40% vs 19%, *P *=* *0.282).

**Table 2.** Comparison of therapeutic efficacy and adverse effects

Outcome	Age ≥65 years	Age <65 years	Odds ratio (95% CI)	*P*-value
	(*n* = 25)	(*n* = 100)		
Endoscopic mucosal healing	5/10 (50%)	27/51 (53%)	0.65 (0.19–2.30)	0.507
Resolution of inflammation on CT/MR enterography	1/10 (10%)	8/53 (15%)	0.45 (0.03–6.33)	0.551
IBD-related surgery	6/15 (40%)	10/53 (19%)	2.39 (0.49–11.65)	0.282
Arthralgia	1/8 (12%)	1/50 (2%)	–*	0.681
Rash	2/14 (14%)	6/51 (12%)	0.44 (0.03–6.57)	0.553
Dyspnea	2/14 (14%)	3/51 (6%)	0.82 (0.04–17.32)	0.897
Infusion reactions	1/24 (4%)	1/96 (1%)	4.00 (0.25–63.95)	0.327
Infections	1/24 (4%)	10/96 (10%)	0.29 (0.03–3.05)	0.300

CI, confidence interval; IBD, inflammatory bowel disease.

* Too few data points in the strata to calculate odds ratio.

**Figure 1.**
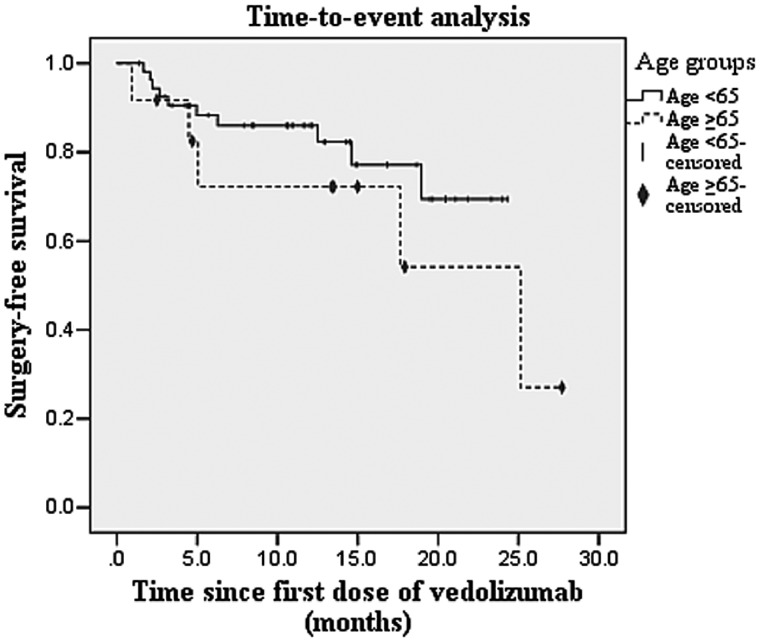
Kaplan–Meier curves comparing surgery-free survival between the elderly (age ≥65 years) and younger (age <65 years) groups

Using Kaplan–Meier analysis, the time to surgery on vedolizumab was found to be comparable between the two groups (*P *=* *0.346; Figure 1). Using logistic-regression analyses, none of the baseline factors examined, including age, gender, body mass index, type of IBD and its phenotype, prior IBD therapies, endoscopic severity, or baseline C-reactive protein, was found to be predictive of increased risk of surgery among the elderly patients on vedolizumab. Using a Cox regression model, none of these factors was found to be predictive of time to IBD-related surgery.

### Adverse effects

Overall, vedolizumab was well tolerated among our study subjects. The most commonly reported adverse effects in the elderly group were skin rash (14%) and shortness of breath (14%), while rash (12%) and infections (10%) were most common among the younger group. No treatment-related deaths were reported. The incidence of the most frequent vedolizumab-related adverse effects—rash, arthralgia, infections, infusion reactions, and dyspnea—were all comparable between the two groups (all *P *>* *0.05) (Table 2), suggesting that the agent has a good safety profile among the elderly.

## Discussion

In this study, we compared the therapeutic efficacy and toxicities of vedolizumab in elderly (≥65 years) IBD patients to matched controls (IBD patients <65 years of age). The two groups were similar at baseline with regard to disease phenotype and previous therapies, including exposure to anti-TNFα drugs. The majority of our patients had previously received anti-TNFα therapy (78% among the elderly and 91% among the younger group) and had undergone some IBD-related bowel surgery (60% of the elderly vs 63% of controls). Vedolizumab was shown to have efficacy among elderly patients, in promoting mucosal healing and averting surgery that was comparable to younger controls. Mucosal healing was assessed using endoscopy (defined by a Mayo Clinic Endoscopic Subscore ≤1 in UC or the resolution of ulceration in CD). Though the older group trended to require more IBD-related surgery, the difference did not reach statistical significance. Vedolizumab was well tolerated with comparable adverse-effect profiles among the groups. The findings of our study suggest that vedolizumab is a safe and effective option among elderly IBD patients, including those who have been pre-treated with anti-TNFα agents.

A significant proportion of patients with IBD are elderly, either with long-standing disease or late presentation [1–5]. Management of these patients is complicated by the lack of good-quality evidence to guide treatment decisions, higher prevalence of co-morbidities, and risk of immunosuppression-related complications [6–9]. Elderly patients have been underrepresented in most IBD drug trials, including those for vedolizumab, in part due to stringent exclusion criteria and intensity of follow-up. In the GEMINI 1 and GEMINI 2 trials, only 4% and 2% of patients, respectively, were ≥65 years old [13, 14]. Similarly, the average age of participants in most subsequent prospective studies of vedolizumab in real-world settings has ranged from thirties to forties, with very few older than 65 years [16–25]. In early 2017, Yajnik *et al.* [26], based on post-hoc subgroup analyses of data from GEMINI 1 and 2 trials, demonstrated similar clinical efficacy and safety profiles of vedolizumab among patients stratified into three age groups: <35 years, 35 to <55 years, and ≥55 years, with most efficacy outcomes being better with vedolizumab than placebo across the age strata. Though they reported data separately for patients <65 years and ≥65 years old, their study was not powered to compare the safety and efficacy outcomes between these age groups. More recently, Navaneethan *et al.* [27], in a retrospective observational study without a comparison group, described the clinical-remission rates and adverse effects of vedolizumab in 29 IBD patients >60 years (19 with CD and 10 with UC). Our study was the first to directly compare the efficacy and safety of vedolizumab in IBD among elderly patients with their matched younger controls.

The primary efficacy outcome in our study was mucosal healing, which is being increasingly regarded as an important surrogate endpoint both in clinical trials and in the clinical management of IBD. Mucosal healing has previously been shown to be associated with higher rates of steroid-free clinical remission, reduction in the need for IBD-related surgery and hospitalization, and decreased risk of colonic dysplasia [28–31]. Though there is no universal agreement on how to define mucosal healing, it has been suggested that a combination of endoscopic and imaging techniques be used in the assessment. For the purposes of this study, we defined mucosal healing as a Mayo Clinic Endoscopic Subscore of 0/1 in UC or resolution of ulceration in CD on endoscopy. We also assessed the resolution of bowel inflammation seen on baseline CT or MR enterography. Recently, Noman *et al.* [32], using data from a single-center cohort of patients enrolled in the GEMINI LTS trial, showed that vedolizumab induced durable endoscopic healing in 29% of CD and 50% of UC patients. The multicenter US VICTORY (Vedolizumab for Health Outcomes in Inflammatory Bowel Diseases) consortium reported mucosal healing rate of 63% in patients with moderately to severely active CD after vedolizumab maintenance for 1 year [20]. In the GEMINI 1 trial, mucosal healing rates in UC at 52 weeks were 52%–56% vs 20% in the placebo group [13]. However, little is known about mucosal healing rates among elderly patients treated with vedolizumab, since these studies enrolled few to no patients >65 years. Our study showed that endoscopic mucosal healing rates with vedolizumab were similar among elderly patients and their younger controls.

Vedolizumab, by virtue of its gut-selective action, is an appealing alternative in elderly patients who are at increased risk of serious infections and malignancies in the setting of systemic immunosuppression. Colombel *et al.* [33], through integrated analysis of safety data from four phase 3 (GEMINI 1, 2, 3 and LTS) and two phase 2 (NCT01177228 and NCT00619489) clinical trials, demonstrated that incidence rates of all as well as serious adverse events, when adjusted for the duration of exposure, were lower with vedolizumab than with placebo. In their study, the most common adverse events among patients exposed to vedolizumab were nasopharyngitis, abdominal pain, headache, and arthralgia. Less than 1% experienced infections serious enough to discontinue therapy and <5% experienced infusion reactions. No case of progressive multifocal leukoencephalopathy was reported. Data were inconclusive regarding the risk of malignancies [33]. In a combined analysis of post-marketing cohort studies, arthralgia (3%), headache (2%), and arthritis (1%) were the most frequently reported non-infectious adverse events [34]. The most frequent infections reported were upper respiratory (4%) and gastrointestinal (2%). In the age-stratified post-hoc analysis of GEMINI 1 and 2 trials described earlier, there were no significant differences in the rates of adverse events, including infections, between the age groups [26]. However, as previously discussed, all these studies included few patients >65 years, limiting the available evidence regarding the safety of vedolizumab in this population. Our study demonstrated a favorable safety profile for vedolizumab among the elderly, with low rates of infection (4%) and infusion-related reactions (4%). No treatment-related malignancy or mortality was reported and no patient had to discontinue therapy due to serious treatment-related adverse events.

This study demonstrates that, in a real-world out-of-clinical-trial setting, vedolizumab is effective in inducing and maintaining mucosal remission among elderly patients, including those pre-treated with anti-TNFα drugs. The rate of IBD-related surgery in this high-risk population was not increased when compared to younger controls. This study also showed that the safety profile of vedolizumab among elderly IBD patients was as favorable as previously demonstrated among younger patients who were enrolled in various clinical trials. These findings suggest that vedolizumab is an appealing treatment option among elderly patients with moderately to severely active IBD.

Our study had several limitations. Mucosal healing on endoscopy was used as a surrogate endpoint while histological healing and clinical-remission rates were not assessed. Capsule endoscopy and push enteroscopy are not routinely used to assess mucosal healing in IBD at our institution, hence healing rates as assessed by these modalities could not be assessed in this retrospective study. This study was not designed to assess the risk of colonic dysplasia or malignancy. The sample size was relatively small, particularly for matched strata-wise comparisons, resulting in wide confidence intervals for calculated odds ratios. Enrollment was not randomized and data were collected retrospectively. Patient inclusion and analysis of outcomes were not based on the type of IBD.

In conclusion, vedolizumab was shown in this study to have comparable efficacy among elderly patients as in younger controls in leading to mucosal healing and averting IBD-related surgery. Vedolizumab was also demonstrated to have a favorable safety profile among elderly patients with IBD. Larger prospective studies with longer follow-up are needed to further confirm these findings.

## Authors’ contributions

Shashi: Data gathering and manuscript preparation; Gopalakrishnan: Statistical analysis, manuscript review and critical revision; Parikh: Data gathering and manuscript review; Shen: Performance of the endoscopic procedures, manuscript review, and critical revision; Kochhar: Study concept, manuscript review and critical revision.

## Funding

This work was not supported by any funding source.

## References

[1] Kappelman MD, Moore KR, Allen JK et al Recent trends in the prevalence of Crohn's disease and ulcerative colitis in a commercially insured US population. Dig Dis Sci 2013;58:519–25. 22926499 10.1007/s10620-012-2371-5 PMC3576554

[2] Lakatos PL, David G, Pandur T et al IBD in the elderly population: results from a population-based study in Western Hungary, 1977–2008. J Crohns Colitis 2011;5:5–13. 21272797 10.1016/j.crohns.2010.08.004

[3] Loftus CG, Loftus EV Jr, Harmsen WS et al Update on the incidence and prevalence of Crohn's disease and ulcerative colitis in Olmsted County, Minnesota, 1940–2000. Inflamm Bowel Dis 2007;13:254–61. 17206702 10.1002/ibd.20029

[4] Taleban S, Colombel JF, Mohler MJ et al Inflammatory bowel disease and the elderly: a review. J Crohns Colitis 2015;9:507–15. 25870198 10.1093/ecco-jcc/jjv059

[5] Navaneethan U, Zhu X, Lourdusamy D et al Colorectal cancer resection rates in patients with inflammatory bowel disease: a population-based study. Gastroenterol Rep (Oxf) 2018;64:263–9. 10.1093/gastro/goy030 PMC6225820 30430014

[6] Manosa M, Calafat M, de Francisco R et al Phenotype and natural history of elderly onset inflammatory bowel disease: a multicentre, case-control study. Aliment Pharmacol Ther 2018;47:605–14. 29369387 10.1111/apt.14494

[7] Ananthakrishnan AN, Donaldson T, Lasch K et al Management of inflammatory bowel disease in the elderly patient: challenges and opportunities. Inflamm Bowel Dis 2017;23:882–93. 28375885 10.1097/MIB.0000000000001099 PMC5687915

[8] Taleban S, Elquza E, Gower-Rousseau C et al Cancer and inflammatory bowel disease in the elderly. Dig Liver Dis 2016;48:1105–11. 27289334 10.1016/j.dld.2016.05.006

[9] John ES, Katz K, Saxena M et al Management of inflammatory bowel disease in the elderly. Curr Treat Options Gastroenterol 2016;14:285–304. 27387455 10.1007/s11938-016-0099-6

[10] Haanstra KG, Hofman SO, Lopes Estevao DM et al Antagonizing the alpha4beta1 integrin, but not alpha4beta7, inhibits leukocytic infiltration of the central nervous system in rhesus monkey experimental autoimmune encephalomyelitis. J Immunol 2013;190:1961–73. 23365083 10.4049/jimmunol.1202490

[11] Fedyk ER, Wyant T, Yang LL et al Exclusive antagonism of the alpha4 beta7 integrin by vedolizumab confirms the gut-selectivity of this pathway in primates. Inflamm Bowel Dis 2012;18:2107–19. 22419649 10.1002/ibd.22940

[12] Soler D, Chapman T, Yang LL et al The binding specificity and selective antagonism of vedolizumab, an anti-alpha4beta7 integrin therapeutic antibody in development for inflammatory bowel diseases. J Pharmacol Exp Ther 2009;330:864–75. 19509315 10.1124/jpet.109.153973

[13] Feagan BG, Rutgeerts P, Sands BE et al Vedolizumab as induction and maintenance therapy for ulcerative colitis. N Engl J Med 2013;369:699–710. 23964932 10.1056/NEJMoa1215734

[14] Sandborn WJ, Feagan BG, Rutgeerts P et al Vedolizumab as induction and maintenance therapy for Crohn's disease. N Engl J Med 2013;369:711–21. 23964933 10.1056/NEJMoa1215739

[15] Schroeder KW, Tremaine WJ, Ilstrup DM. Coated oral 5-aminosalicylic acid therapy for mildly to moderately active ulcerative colitis: a randomized study. N Engl J Med 1987;317:1625–9. 3317057 10.1056/NEJM198712243172603

[16] Samaan MA, Pavlidis P, Johnston E et al Vedolizumab: early experience and medium-term outcomes from two UK tertiary IBD centres. Frontline Gastroenterol 2017;8:196–202. 28839909 10.1136/flgastro-2016-100720 PMC5558276

[17] Stallmach A, Langbein C, Atreya R et al Vedolizumab provides clinical benefit over 1 year in patients with active inflammatory bowel disease-a prospective multicenter observational study. Aliment Pharmacol Ther 2016;44:1199–212. 27714831 10.1111/apt.13813

[18] Baumgart DC, Bokemeyer B, Drabik A et al Vedolizumab induction therapy for inflammatory bowel disease in clinical practice--a nationwide consecutive German cohort study. Aliment Pharmacol Ther 2016;43:1090–102. 27038247 10.1111/apt.13594

[19] Vivio EE, Kanuri N, Gilbertsen JJ et al Vedolizumab effectiveness and safety over the first year of use in an IBD clinical practice. ECCOJC 2016;10:402–9. 10.1093/ecco-jcc/jjv226 PMC4946762 26681763

[20] Dulai PS, Singh S, Jiang X et al The real-world effectiveness and safety of vedolizumab for moderate-severe Crohn's disease: results from the US VICTORY Consortium. Am J Gastroenterol 2016;111:1147–55. 27296941 10.1038/ajg.2016.236

[21] Wang MC, Zhang LY, Han W et al PRISMA--efficacy and safety of vedolizumab for inflammatory bowel diseases: a systematic review and meta-analysis of randomized controlled trials. Medicine (Baltimore) 2014;93: e326. 25526490 10.1097/MD.0000000000000326 PMC4603082

[22] Amiot A, Serrero M, Peyrin-Biroulet L et al One-year effectiveness and safety of vedolizumab therapy for inflammatory bowel disease: a prospective multicentre cohort study. Aliment Pharmacol Ther 2017;46:310–21. 28593685 10.1111/apt.14167

[23] Feagan BG, Rubin DT, Danese S et al Efficacy of vedolizumab induction and maintenance therapy in patients with ulcerative colitis, regardless of prior exposure to tumor necrosis factor antagonists. Clin Gastroenterol Hepatol 2017;15:229–39.e5. 27639327 10.1016/j.cgh.2016.08.044

[24] Shelton E, Allegretti JR, Stevens B et al Efficacy of vedolizumab as induction therapy in refractory IBD patients: a multicenter cohort. Inflamm Bowel Dis 2015;21:2879–85. 26288002 10.1097/MIB.0000000000000561 PMC4745906

[25] Kopylov U, Ron Y, Avni-Biron I et al Efficacy and safety of vedolizumab for induction of remission in inflammatory bowel disease--the Israeli real-world experience. Inflamm Bowel Dis 2017;23:404–8. 28178003 10.1097/MIB.0000000000001039

[26] Yajnik V, Khan N, Dubinsky M et al Efficacy and safety of vedolizumab in ulcerative colitis and Crohn's disease patients stratified by age. Adv Ther 2017;34:542–59. 28070861 10.1007/s12325-016-0467-6 PMC5331094

[27] Navaneethan U, Edminister T, Zhu X et al Vedolizumab is safe and effective in elderly patients with inflammatory bowel disease. Inflamm Bowel Dis 2017;23:E17. 28296827 10.1097/MIB.0000000000001071

[28] Rutter M, Saunders B, Wilkinson K et al Severity of inflammation is a risk factor for colorectal neoplasia in ulcerative colitis. Gastroenterology 2004;126:451–9. 14762782 10.1053/j.gastro.2003.11.010

[29] Schnitzler F, Fidder H, Ferrante M et al Mucosal healing predicts long-term outcome of maintenance therapy with infliximab in Crohn's disease. Inflamm Bowel Dis 2009;15:1295–301. 19340881 10.1002/ibd.20927

[30] Neurath MF, Travis SP. Mucosal healing in inflammatory bowel diseases: a systematic review. Gut 2012;61:1619–35. 22842618 10.1136/gutjnl-2012-302830

[31] Colombel JF, Rutgeerts PJ, Sandborn WJ et al Adalimumab induces deep remission in patients with Crohn's disease. Clin Gastroenterol Hepatol 2014;12:414–22.e5. 23856361 10.1016/j.cgh.2013.06.019

[32] Noman M, Ferrante M, Bisschops R et al Vedolizumab induces long-term mucosal healing in patients with Crohn's disease and ulcerative colitis. J Crohns Colitis 2017;11:1085–9. 28369329 10.1093/ecco-jcc/jjx048

[33] Colombel JF, Sands BE, Rutgeerts P et al The safety of vedolizumab for ulcerative colitis and Crohn's disease. Gut 2017;66:839–51. 26893500 10.1136/gutjnl-2015-311079 PMC5531223

[34] Bye WA, Jairath V, Travis SPL. Systematic review: the safety of vedolizumab for the treatment of inflammatory bowel disease. Aliment Pharmacol Ther 2017;46:3–15. 28449273 10.1111/apt.14075

